# When double-J stents become a risk: bilateral distal ureteric urothelial carcinoma following ureteric instrumentation: a case report

**DOI:** 10.1186/s12894-026-02183-5

**Published:** 2026-05-22

**Authors:** Salma Eldesouki, Amr Elmekresh, Hamzeh Esmaeilpour, Senthil Kumar, Khaldon Abo Alsel, Elham Mahjoor Azad, Fariborz Bagheri

**Affiliations:** 1 Department of Urology, Dubai Hospital, Mohamed Bin Rashid University of Medicine and Health Sciences, Dubai, United Arab Emirates; 2 Department of Family Medicine, Medcare Hospital, Dubai, United Arab Emirates

**Keywords:** Upper tract urothelial carcinoma, Metachronous tumor, Ureteric stent, Urothelial carcinoma, Non-muscle-invasive bladder cancer, Urinary diversion

## Abstract

**Background:**

Metachronous upper tract urothelial carcinoma (UTUC) is an uncommon but clinically significant event in patients with non–muscle-invasive bladder cancer (NMIBC), particularly in those with high-risk or recurrent disease. Proposed mechanisms include field cancerization and intraluminal tumor seeding, while ureteric instrumentation has been discussed as a potential contributing factor. Emerging evidence suggests that retrograde ureteric stent placement performed concurrently with transurethral resection of bladder tumor (TURBT) may be associated with an increased risk of subsequent upper tract involvement. We report the first case of bilateral distal ureteric urothelial carcinoma developing in close temporal association with first-time ureteric stent placement in a patient with recurrent NMIBC.

**Case report:**

A 78-year-old man with recurrent high-grade NMIBC underwent bilateral ureteric stent placement during TURBT for obstructive uropathy, with no prior evidence of UTUC. Within months, imaging demonstrated progressive distal ureteric abnormalities. Subsequent cystoscopy and bilateral ureteroscopy revealed extensive papillary tumors involving both distal ureters with proximal extension, in addition to recurrent bladder lesions. Histopathology confirmed high-grade, non–muscle-invasive urothelial carcinoma. At re-intervention, both indwelling stents were encased within tumor tissue.

**Conclusion:**

This case highlights the multifactorial nature of metachronous UTUC in patients with recurrent bladder cancer and supports the hypothesis that ureteric instrumentation may contribute to upper tract tumor dissemination in selected high-risk settings. Careful consideration of urinary diversion strategies and close upper tract surveillance remain important when managing patients with active bladder urothelial carcinoma.

## Background

Urothelial carcinoma (UC) arises from the urothelial lining of the urinary tract and most often involves the bladder, accounting for more than 90% of all cases [1, 2]. It predominantly affects older males and has been closely linked to cigarette smoking [3]. The disease often presents insidiously, with painless hematuria being the most common manifestation, occasionally accompanied by irritative lower urinary tract symptoms [1].

Urothelial carcinoma of the bladder is broadly classified based on depth of invasion into non–muscle-invasive bladder cancer (NMIBC) and muscle-invasive bladder cancer (MIBC) [4]. NMIBC encompasses tumors limited to the mucosa (Ta), lamina propria (T1), or carcinoma in situ (CIS), and represents approximately 75% of new diagnoses [2]. Despite being non-invasive, it is characterized by its recurrent nature, with reported five-year recurrence rates ranging from 31% to 78%, and progression risks approaching 40–50% in selected high- and very-high-risk cases [5]. Recurrence and progression are influenced by tumor grade, size, multifocality, stage, and the presence of CIS.

By contrast, MIBC invades the detrusor muscle (stage T2 or higher) and accounts for about 25% of new UC diagnoses [4]. It carries a substantially higher risk of metastasis and disease-specific mortality and typically requires radical cystectomy or multimodal therapy [1]. Long-term outcome data indicate that a substantial proportion of patients with high-risk NMIBC may ultimately progress to muscle-invasive disease despite intravesical therapy [2].

A subset of patients with UC subsequently develop metachronous upper tract urothelial carcinoma (UTUC), defined as a new urothelial malignancy of the renal pelvis or ureter occurring after an initial diagnosis of bladder UC [6]. Recent multi-institutional cohort data demonstrate that following NMIBC, the 10-year cumulative incidence of metachronous UTUC is approximately 2.2% in low-risk patients, 4.4% in intermediate-risk patients, and 6.3% in those with high- and very-high-risk disease, with the majority of cases (approximately 77%) diagnosed within the first five years after the primary bladder tumor [7]. The risk is significantly higher among patients with high-grade, recurrent, or multifocal bladder UC, as well as in those with tumors involving the trigone or ureteric orifice.

Several mechanisms have been proposed to explain the development of metachronous UTUC following bladder urothelial carcinoma, including field cancerization and intraluminal seeding, whereby malignant cells disseminate along the urothelial lining [7]. Molecular studies have demonstrated that both processes may contribute to disease evolution, with many synchronous and metachronous bladder and upper tract tumors showing clonal relatedness [8–12]. In addition to these intrinsic mechanisms, ureteric instrumentation has been increasingly associated with an elevated risk of subsequent upper tract recurrence in patients with bladder urothelial carcinoma [13].

Against this background, we present a case of an elderly man with long-standing non–muscle-invasive bladder cancer who developed bilateral distal ureteric urothelial carcinoma following first-time ureteric stent placement. To our knowledge, this pattern of bilateral distal ureteric involvement in close temporal association with stent insertion has not been previously reported. This case provides an opportunity to examine potential mechanistic and clinical considerations surrounding ureteric instrumentation in patients with active bladder urothelial carcinoma.

## Case presentation

### Initial diagnosis and treatment

Mr. M.K., a 78-year-old Middle Eastern gentleman, presented to the urology clinic at Dubai Hospital for the first time in December 2024 with a comprehensive set of medical reports from several private hospitals documenting a long-standing history of non–muscle-invasive urothelial carcinoma (NMIBC) of the bladder. His medical history was notable only for hypertension, well controlled on amlodipine 5 mg and valsartan 160 mg daily. He had never smoked, had no known occupational exposure to urothelial carcinogens, and reported no family history of malignancy. Physical examination was unremarkable, with no palpable abdominal or flank masses and no suprapubic tenderness.

According to the submitted records, his illness began in 2018 with a transurethral resection of bladder tumour (TURBT) confirming papillary urothelial carcinoma, pTa. He completed induction and maintenance of intravesical Bacillus Calmette–Guérin (BCG) therapy over one year, with no early recurrence. Between 2019 and 2020, he underwent multiple TURBTs for recurrent low-grade and later high-grade non-invasive disease. By late 2020, cystoscopy demonstrated extensive multifocal papillary lesions reaching the prostatic urethra. Internal urethrotomy and TURBT were performed, revealing high-grade pTa carcinoma. Cross-sectional imaging showed no metastatic disease. A second induction course of BCG was initiated in December 2020, after which he was lost to follow-up.

### Disease progression and upper tract involvement

He re-presented in mid-2024 to a private facility with symptoms of bladder outlet obstruction. Imaging demonstrated bilateral hydronephrosis, a markedly contracted bladder, and diffuse bladder wall thickening with possible extravesical extension. A positron emission tomography (PET) scan performed as part of the same workup showed a mildly enlarged, metabolically active left femoral lymph node. Radical cystectomy was recommended but not performed. Seeking further evaluation, he attended Dubai Hospital later that year with all prior imaging and histopathology. Following multidisciplinary review, active surveillance was chosen in the absence of unequivocal muscle invasion or distant metastasis.

In December 2024, he underwent cystoscopy, cystography, bilateral retrograde pyelography, and TURBT with first-time bilateral ureteric stent placement in Dubai Hospital. The bladder was markedly contracted, with an estimated capacity of approximately 10 mL, and multiple papillary lesions were noted along the posterior wall, dome, and left ureteric orifice. Bilateral retrograde pyelography demonstrated no filling defects or intraluminal abnormalities within the ureters. Histopathology unexpectedly demonstrated polypoid and papillary cystitis without evidence of malignancy, despite his extensive history of recurrent high-grade disease.

**Fig. 1:**
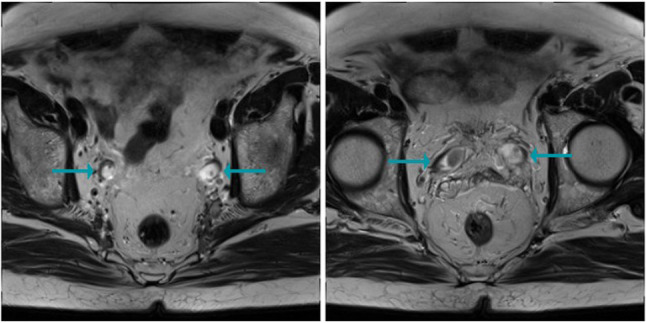
Bilateral distal ureteric filling defects on pelvic MRI. Axial pelvic MRI images demonstrating bilateral, symmetric intraluminal filling defects within the distal ureters (arrows), resulting in partial luminal narrowing

At that stage, no definite intraluminal obstruction was identified on imaging. The hydronephrosis was therefore presumed to be multifactorial, possibly related to bladder outlet obstruction, reduced bladder compliance, and early distal ureteric changes not yet radiographically apparent. His course evolved further over the following months. A pelvic MRI in June 2025 showed a fibrotic, contracted bladder, irregular thickening of both distal ureters, and a small soft-tissue nodule near the right vesicoureteric junction (Fig. 1). The left femoral lymph node remained concerning.

By November 2025, repeat endoscopic evaluation was performed. Cystoscopy and bilateral ureteroscopy revealed new papillary tumors within both distal ureters in addition to recurrent bladder lesions. Intraoperatively, a tight bulbar urethral narrowing was noted, with small papillary lesions along the urethra. The bladder neck and trigone harbored extensive papillary and sessile-appearing tumors, and both previously placed ureteric stents were found to be embedded within tumor tissue. On the right, a large mid-ureteric lesion prevented advancement of the flexible ureteroscope beyond this level toward the renal pelvis. On the left, papillary tumors extended from the distal ureter proximally to the renal pelvis. After tumor resection and removal of the existing stents, bilateral ureteric stents were reinserted over guidewires, with bilateral 7 French gauge × 28 cm double-J stents positioned at the conclusion of the procedure.

Histopathology from the combined bladder and distal ureteric specimens confirmed high-grade papillary urothelial carcinoma (pTa) with no lamina propria invasion. Detrusor muscle was present and uninvolved. At the time of initial detection of bilateral hydronephrosis, no imaging or selective upper tract cytology was performed. This decision was influenced by the absence of radiographic filling defects on retrograde pyelography and the clinical prioritization of bladder pathology. However, in retrospect, earlier upper tract evaluation may have facilitated earlier detection of ureteric involvement.

A repeat PET-CT performed later in November 2025 demonstrated persistent left hydronephrosis and interval enlargement of the previously Fluorodeoxyglucose (FDG)-avid left femoral lymph node. Given its progression, ultrasound-guided biopsy was performed. Histopathology revealed leiomyosarcoma, representing a second, unrelated soft-tissue malignancy rather than metastatic urothelial carcinoma. He was subsequently referred to General Surgery for definitive management of the leiomyosarcoma. The patient is currently planned for multidisciplinary reassessment.

## Discussion

The patient had a long-standing history of recurrent non–muscle-invasive urothelial carcinoma, with multiple prior TURBTs, high-grade disease, multifocality, and chronic bladder inflammation, all established risk factors for UTUC. After several years without documented upper tract involvement, he developed bilateral distal ureteric tumors shortly after first-time ureteric stent insertion. His subsequent evaluation revealed extensive, predominantly non-invasive papillary disease within both ureters, raising the important question of whether this pattern represents natural disease progression or iatrogenic tumor dissemination in a high-risk anatomical and biological setting. To our knowledge, this is the first reported case describing bilateral distal ureteric urothelial carcinoma developing in close temporal association with ureteric stent placement.

In recent years, en bloc resection of bladder tumor (ERBT) has emerged as an alternative to conventional transurethral resection (TURBT), aiming to improve specimen quality and staging accuracy [14]. ERBT allows intact tumor removal with preserved architecture, facilitating more accurate assessment of tumor depth and margin status. Despite these advantages, its adoption remains limited by technical challenges, tumor size, location, and the need for specialized equipment. Furthermore, evidence regarding its impact on long-term oncologic outcomes, including recurrence and progression rates, is still evolving.

Repeated transurethral resections and intravesical Bacillus Calmette–Guérin (BCG) therapy may also have contributed to progressive bladder fibrosis and reduced bladder capacity in this patient. The markedly contracted bladder (approximately 10 mL capacity) is most consistent with cumulative treatment-related fibrosis from multiple TURBTs and prior BCG exposure rather than a single isolated event. Chronic inflammation induced by BCG and repeated instrumentation has been associated with a contracted, low-compliance bladder, which may further alter intravesical dynamics.

**Fig. 2:**
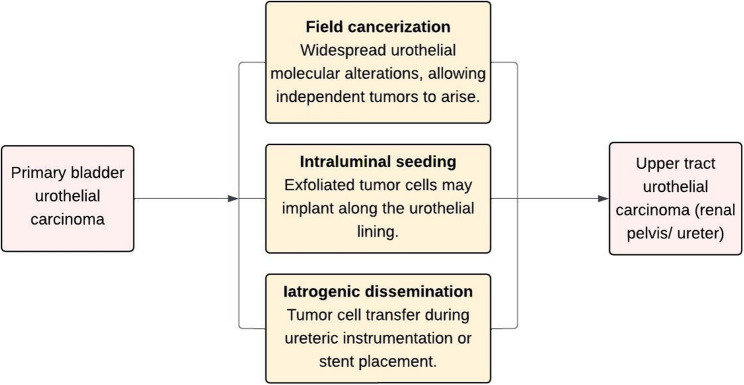
Proposed mechanisms of metachronous upper tract urothelial carcinoma. Schematic overview of the main mechanisms proposed to explain the development of metachronous UTUC after bladder urothelial carcinoma, including field cancerization, intraluminal seeding, and tumor dissemination related to ureteric instrumentation

The development of UTUC in patients with longstanding bladder cancer is thought to occur through more than one biological pathway (Fig. 2). While some cases reflect de novo tumorigenesis within a chronically altered urothelial field, others appear to arise from dissemination of a single malignant clone along the urinary tract. Distinguishing between these mechanisms is particularly relevant when interpreting atypical patterns of disease progression.

Historically, multifocal urothelial tumors were most commonly attributed to field cancerization, a model proposing that prolonged carcinogenic injury to the urothelium creates a biologically altered field in which tumors may arise independently at different sites over time [12]. Early molecular studies using loss-of-heterozygosity analysis and X-chromosome inactivation patterns demonstrated genetic discordance among synchronous and metachronous urothelial tumors within the same patient, findings consistent with oligoclonality and independent tumorigenesis rather than direct spread from a single lesion [12, 15]. These observations reinforced the view that upper tract involvement frequently represents a de novo process arising within a globally altered urothelial field.

With the advent of next-generation sequencing, this framework has been refined. More recent molecular analyses comparing paired bladder and upper tract tumors have demonstrated that synchronous and metachronous lesions are frequently clonally related, sharing key driver mutations and truncal genomic alterations. In a landmark study, Audenet et al. demonstrated that upper tract and bladder urothelial carcinomas arising in the same patient were often derived from a common ancestral clone, supporting monoclonal dissemination as a dominant mechanism in many cases [10]. Subsequent systematic and multi-omic studies have confirmed that field cancerization and monoclonal spread are not mutually exclusive, with their relative contribution varying according to tumor biology and clinical context [8, 11]. In general, field effects appear more prominent in early or low-grade multifocal disease, whereas monoclonal spread predominates in recurrent, high-risk, or anatomically contiguous tumors.

In the present case, several features argue against classical field cancerization as the primary mechanism of upper tract involvement. Despite a long-standing history of high-risk non–muscle-invasive urothelial carcinoma, the patient had no documented upper tract disease for several years, followed by the abrupt and near-simultaneous development of bilateral distal ureteric tumors. This pattern is difficult to explain by independent tumor development alone. Field cancerization would more typically result in upper tract tumors that appear over time and with some degree of heterogeneity. Instead, the bilateral, near-simultaneous involvement of the distal ureters with similar papillary, non–muscle-invasive histology points toward a common underlying process.

An alternative explanation that must be considered is the possibility of metachronous UTUC developing independently during the period when the patient was lost to follow-up. Given the four-year gap in surveillance, occult upper tract disease may have arisen prior to stent placement but remained undetected on subsequent imaging. Therefore, while the temporal association with ureteric instrumentation is notable, a coincidental progression of high-risk urothelial disease cannot be definitively excluded.

Alternatively, another explanation is iatrogenic tumor dissemination temporally associated with ureteric instrumentation. A systematic review and meta-analysis demonstrated an increased risk of metachronous UTUC following retrograde ureteric stent placement in patients with bladder cancer, with reported odds ratios ranging from 3.07 to 4.54 [9]. Proposed mechanisms include mechanical transfer of malignant cells during instrumentation and retrograde migration of tumor cells along the stent surface [13]. Importantly, a retrospective cohort study identified double-J stent insertion performed at the time of TURBT as an independent risk factor for subsequent UTUC on multivariable analysis, suggesting that instrumentation in the setting of active bladder tumor may facilitate retrograde tumor cell dissemination [16].

While several studies have reported a relatively low overall incidence of metachronous UTUCa following ureteric stent placement, these analyses generally evaluate stenting as a broad exposure across diverse clinical settings [17, 18]. In many cases, stents are inserted for varying indications and at different time points, without consistent distinction between placement in the absence of active disease and insertion performed during ongoing bladder tumor resection. Consequently, the reported incidence reflects average population risk rather than the procedural risk associated with stent placement in the setting of active intravesical tumor manipulation. In this context, the finding from a study examining retrograde stent insertion performed concurrently with TURBT suggests that both the timing of stent placement and the presence of active tumor may be key modifiers of risk [16].

In addition, secondary vesicoureteral reflux (VUR) may represent another contributing mechanism. A markedly contracted bladder, as observed in this case, can increase intravesical pressure and predispose to reflux of urine and exfoliated tumor cells into the upper urinary tract [19]. Although VUR has not been established as an independent risk factor for UTUC, it may facilitate retrograde dissemination in the presence of active urothelial carcinoma, particularly when combined with ureteric instrumentation.

In this patient, repeated CT imaging and retrograde evaluations after prior TURBT procedures revealed no upper tract lesions, despite probable reduced bladder capacity and underlying reflux mechanisms. This makes reflux alone an unlikely explanation for the observed pattern. Rather, it may have acted as a facilitating factor in combination with mechanical effects introduced by ureteric stents, contributing to intraluminal tumor dissemination in a high-risk setting.

This procedural context closely parallels the present case. The patient underwent first-time bilateral ureteric stent insertion during TURBT in a bladder with active papillary disease, after several years without radiographic or endoscopic evidence of upper tract involvement. The subsequent development of extensive distal ureteric tumors, together with intraoperative findings of tumor encasing the indwelling stents, provides direct anatomical support for stent-associated intraluminal tumor migration. While causality cannot be definitively established, the temporal relationship and disease distribution strongly support iatrogenic seeding as a plausible contributing factor in this case, rather than coincidental de novo tumorigenesis.

Evidence from observational studies comparing diversion strategies has yielded mixed results. Both retrograde ureteric stenting and percutaneous nephrostomy (PCN) are effective options for upper tract decompression. Earlier retrospective series suggested a higher risk of metachronous UTUC following ureteric stent placement compared with nephrostomy, raising concern that retrograde instrumentation may facilitate tumor cell dissemination [10, 16]. However, more recent multicenter analyses and meta-analyses have not demonstrated a statistically significant difference in upper tract recurrence, cancer-specific survival, or overall survival after adjustment for confounders, although the overall quality of evidence remains limited [13].

When oncologic outcomes between diversion strategies remain uncertain, the choice of urinary diversion is often driven by procedural considerations and patient-centered factors. Comparative studies in malignant ureteral obstruction suggest that percutaneous nephrostomy is associated with higher technical success and longer intervention durability, whereas retrograde ureteric stenting is commonly favored for its shorter procedure time, reduced hospital stay, and avoidance of an external drainage device [20]. Rates of infection, obstruction, and unplanned exchange appear broadly comparable between the two approaches, while quality-of-life outcomes and patient preferences vary across studies [21, 22]. Taken together, this body of literature highlights the heterogeneity of malignant obstruction scenarios and supports an individualized, case-by-case approach to diversion selection, particularly in patients with active pelvic malignancy.

Reflecting this uncertainty, the European Association of Urology (EAU) guidelines do not recommend one diversion modality over the other on oncologic grounds and emphasize that the choice between ureteric stenting and percutaneous nephrostomy should be individualized based on clinical context, tumor location, anatomical considerations, and technical feasibility [6]. Similarly, the American College of Radiology (ACR) Appropriateness Criteria recognize both approaches as acceptable for malignant urinary obstruction and do not identify a clear difference in oncologic outcomes between the two modalities [23]. In patients with active bladder urothelial carcinoma, particularly when the trigone or ureteric orifices are involved, the potential oncologic implications of retrograde instrumentation should be carefully balanced against its procedural advantages.

In general, new surgical methods like ERBT may have an impact on NMIBC early care and staging [24]. Improved en bloc specimen extraction could help identify high-risk characteristics and direct more accurate risk assessment early on. Although findings are currently inconsistent and long-term comparisons with traditional TURBT are still being studied, several research indicate possible decreases in residual tumor and recurrence rates [24]. Future studies should concentrate on patient selection, the adoption of standardized techniques, and if early ERBT use could lessen downstream consequences, such as upper tract involvement in high-risk instances.

In theory, stent designs that limit vesicoureteral reflux, such as anti-reflux ureteric stents, could reduce retrograde exposure of the upper tract to exfoliated tumor cells. These designs have been shown to reduce vesicoureteral reflux and stent-related symptoms compared with standard double-J stents, without compromising safety [25]. However, there is currently no clinical evidence that they influence tumor dissemination or oncologic outcomes, and this remains a promising area for future study.

The combination of active tumor manipulation during TURBT and the presence of ureteric stents may create conditions conducive to retrograde tumor cell dissemination, particularly in a high-risk bladder environment. Stent designs that limit vesicoureteral reflux, such as anti-reflux ureteric stents, represent a potential strategy to reduce retrograde exposure of the upper tract to tumor cells. However, their impact on oncologic outcomes remains unproven and warrants further investigation.

## Conclusion

This case highlights the need for careful decision-making when urinary diversion is required in patients with active bladder urothelial carcinoma. In high-risk settings, particularly when the trigone or ureteric orifices are involved, the potential oncologic implications of retrograde ureteric instrumentation should be weighed against its clinical benefits, and close upper tract surveillance remains essential. In the present case, the bilateral and distal pattern of ureteric involvement supports a mechanism of intraluminal tumor dissemination facilitated by retrograde exposure of the upper tract. Further studies are needed to better define the role of ureteric instrumentation in this context and to clarify potential strategies to mitigate associated risks.

## Data Availability

No datasets were generated or analysed during the current study.

## Abbreviations

UC: Urothelial carcinoma
NMIBC: Non—muscle-invasive bladder cancer
MIBC: Muscle-invasive bladder cancer
CIS: Carcinoma in situ
UTUC: Upper tract urothelial carcinoma
TURBT: Transurethral resection of bladder tumour
BCG: Bacillus Calmette—Guérin
pTa: Pathologic stage Ta (non—invasive papillary urothelial carcinoma)
PET: Positron emission tomography
MRI: Magnetic resonance imaging
FDG: Fluorodeoxyglucose
CT: Computed tomography
PET: Positron emission tomography
PCN: Percutaneous nephrostomy
EAU: European Association of Urology
ACR: American College of Radiology
